# Development of a 3D-printable bioactive polycaprolactone–collagen peptides filament for biomedical applications

**DOI:** 10.1038/s41598-025-28030-5

**Published:** 2025-12-24

**Authors:** Stefano Cantella, Silvia Badini, Carlotta Bollati, Mushtaq Alam Madar Saheb, Roberto Viganò, Carmen Lammi, Raffaele Pugliese, Serena Graziosi

**Affiliations:** 1 School of Industrial and Information Engineering, Politecnico di Milano, 20133, Milan, Italy; 2 NeMO Lab Research Center, ASST GOM Niguarda Cà Granda Hospital, 20152 Milan, Italy; 3 Department of Pharmaceutical Sciences, University of Milan, 20133 Milan, Italy; 4 Department of Mechanical Engineering, Politecnico di Milano, 20156 Milan, Italy

**Keywords:** 3D printing, Filament fabrication, Bioactive materials, Collagen peptides, Polycaprolactone, Tissue engineering, Mechanical engineering, Biomedical materials

## Abstract

This study presents a scalable, nontoxic method for fabricating a 3D printable polycaprolactone (PCL)-collagen peptides composite filament via solvent-assisted blending and a customized desktop filament extrusion system. Virgin and recycled PCL feedstocks were used to study the matrix characteristics. Scanning electron microscopy, X-ray diffraction, differential scanning calorimetry, thermogravimetric analysis, and intrinsic fluorescence spectroscopy confirmed that the composite maintains PCL’s inherent crystalline and thermal properties. It also exhibits intrinsic bioactive capabilities provided by the collagen peptides. Filaments with diameters suitable for the Fused Filament Fabrication 3D printing were obtained. Tests demonstrated that the recycled matrix and the lab-scale process reduce the tensile modulus of the material. At the same time, collagen peptides enhanced tensile stiffness by creating intermolecular hydrogen bonding with the PCL. The biocompatibility of the composite has also been confirmed, while degradability studies have shown the tunability of the PCL degradation rate. Additionally, examples of 3D-printed scaffolds based on Triply Periodic Minimal Surfaces have been successfully fabricated using the PCL-collagen peptide filament. Therefore, this study demonstrates that integrating collagen peptides into a PCL matrix represents a viable, nontoxic, affordable, and promising approach for developing customized and bioactive implants, scaffolds, and other regenerative medicine applications.

**Supplementary Information:**

The online version contains supplementary material available at 10.1038/s41598-025-28030-5.

## Introduction

The continuous advancement in biomedical and tissue engineering applications underscores the need for novel bioactive and biocompatible materials that integrate and/or interact with biological systems^1^. Developing such materials would contribute to applications in the field of tissue regeneration, wound healing, drug delivery, and personalized medical implants and scaffolding^2–4^. As “modern medicine” shifts toward patient-specific treatments and minimally invasive solutions, the demand for innovative biomaterials that promote cellular interaction, tissue growth, and long-term biocompatibility is becoming increasingly relevant, with significant efforts invested so far, but also with new expectations, for example, in terms of costs and long-lasting capabilities^5–7^.

A contribution towards new material developments, in combination with the possibility of fabricating customized and therefore patient-specific solutions, is being provided by additive manufacturing (AM). In recent years, AM has evolved into a promising production paradigm for addressing challenges in tissue engineering, regenerative medicine, and the development of novel healthcare devices^8–10^. Certainly, thanks to its ability to produce customized and complex architectures, 3D printing has pushed the healthcare industry to design and implement patient-specific prostheses, implants, scaffolds, and wearable devices^11–13^. Indeed, the market size of 3D printing in the healthcare industry is reported to be USD 1.66 billion in 2024, estimated to rise to USD 8.71 billion by 2034, with a Compound Annual Growth Rate (CAGR) of 18.03%^14^ while, the market size for biocompatible 3D printing materials was USD 664.7 million in 2024 and is further estimated to expand at a CAGR of 14.6% by 2030^15^. This can be attributed to the growing interest of the healthcare industry in personalized medicines, which may facilitate the development of cost-effective and patient-specific medications, implants, and medical equipment^16,17^. As a result, the scientific community is focusing on developing novel materials that are biocompatible, cost-effective, and enable functional patient-specific solutions^18,19^. However, material availability remains limited, and the costs for their development are still high, hindering their transferability into clinical applications^20,21^.

Despite this rising interest, incorporating bioactive components into polymer matrices for AM processes remains in its early stages. In this context, 3D printable biocompatible polymers such as polylactic acid (PLA), polycaprolactone (PCL), polybutylene adipate terephthalate (PBAT), and polyether ether ketone (PEEK) can be suitable matrices for these components, thanks to their mechanical properties, biodegradability, and Food and Drug Administration (FDA) approved status for biomedical applications. Moreover, these polymers are prone to undergo various chemical and physical modifications, including blending with bioactive molecules, to further enhance their functionality^22–25^. For instance, PLA has already been blended with lignin to create composite materials, processed via injection molding, that exhibit cytocompatibility, antioxidant properties, and hemocompatibility^26^. The fused filament fabrication (FFF) technique has been utilized to 3D print scaffolds with amorphous magnesium phosphate dispersed in a PLA matrix^27^. These scaffolds exhibited enhanced cell attachment and proliferation, along with a faster degradation rate, while a minor decline in mechanical properties was also observed compared to pure PLA^27^. Furthermore, with PLA serving as the matrix, additives such as nano-hydroxyapatite and β-cyclodextrin/chlorhexidine clathrate have also been 3D-printed as porous scaffolds using FFF 3D printing, exhibiting osteogenic and antibacterial properties^28^. PLA has also been successfully combined with biphasic calcium phosphates, exhibiting hydroxyapatite (HA) and tricalcium phosphate (TCP) as the primary inorganic phases, for applications in bone tissue engineering^29^. Instead, for applications in patient-specific craniomaxillofacial implants, zinc- and strontium-doped HA nanoparticles have been mixed with PEEK, resulting in the creation of a novel 3D printable filament^30^. Concerning PVA, over the past few years, the pharmaceutical sector has witnessed the emergence of FFF-based medical capsules utilizing it as the excipient^31^.

However, among the various 3D printable polymeric matrices, PCL stands out as an outstanding candidate due to its exceptional biocompatibility, biodegradability, and compatibility with low-temperature processing^32^. Additionally, as anticipated, it has received FDA approval for safe clinical use^33^. In recent years, novel biomedical applications have been developed using PCL-based composites, and coatings have been applied to enhance the bioactivity of PCL. For example, Farajpour et al.^34^ utilized FFF to blend PCL with human decellularized bone matrix, producing a scaffold that promotes osteogenesis. PCL has also been used to obtain ibuprofen and chitosan-loaded 3D-printable filament to fabricate personalized implants with a sustained drug-releasing profile^35^. Wang et al.^36^ developed 3D-printed PCL-based composite scaffolds with gradient pore geometries, able to provide programmed release of biomolecules to promote osteochondral regeneration^36^. 3D-printable PCL-based composites (PLA/PCL, Poly(3-hydroxybutyrate)/PCL, and nanometric HA/PCL) have also been developed for cardiovascular stents with enhanced cell proliferation rates and the production of scaffolds^37–39^. Additionally, zinc (Zn)-based PCL composites^40^ were used for the fabrication of 3D printing filaments, and a gradual increase in osteogenesis was observed in 3D-printed scaffolds with increasing concentrations of Zn. Temple et al.^41^ induced human adipose-derived stem cells onto a 3D-printed, customized PCL bone graft and found enhanced osteogenic activity. Tabatabaei et al.^42^ presented collagen-coated 3D-printed PCL/TCP scaffolds that exhibited higher water and protein absorption, as well as increased bioactivity, compared to non-coated samples. Similarly, 3D-printed PCL scaffolds coated with collagen type I have exhibited improved cell-scaffold interactions, enhancing cell adhesion and proliferation^43^. Finally, 3D-printed PCL scaffolds were also coated with gold nanoparticles to create electroconductive scaffolds for myocardial tissue regeneration^44^.

Despite these significant advances, very few studies have harnessed the innate properties of biomolecules, such as proteins and peptides, renowned for their exceptional biocompatibility and biodegradability, in the fabrication of 3D-printable materials. This challenge largely stems from the high extrusion temperatures required by thermoplastic matrices, which can denature these molecules^45–48^. Consequently, most research has limited itself to post-processing methods, such as surface coating or the functionalization of 3D-printed artefacts, rather than directly integrating these biomolecules into the polymer matrix to develop truly bioactive composites. Moreover, a thorough investigation of how peptides’ structural and bioactive properties influence the printability, mechanical performance, and morphology of the polymer matrix is still lacking. There is also an absence of clear guidelines for achieving successful, non-toxic blending and fabrication parameters for these bioactive composites.

To address these gaps, this study presents a novel bioactive 3D-printable composite filament obtained by integrating collagen peptides into a PCL matrix using a non-toxic, solvent-assisted blending process. In our method, collagen peptides are first dissolved in a carefully controlled solvent mixture, preserving their bioactivity, and then uniformly blended with the PCL. After drying, the composite material is extruded using a fine-tuned extruder system that produces filaments with a diameter tailored for FFF 3D printing. Therefore, in addition to presenting a new 3D printable and bioactive material, this study also introduces an innovative protocol for non-toxic, solvent-assisted blending and scalable filament fabrication, which preserves the bioactivity of the molecules.

Collagen peptides were selected for their abundance in the extracellular matrix (ECM) and their multiple roles in maintaining tissue health^49^. Being obtained from collagen hydrolysis, these peptides contribute to mechanical strength and structural integrity by promoting tissue development and repair. These peptides are typically obtained through the hydrolysis of animal-derived waste materials, especially those generated by the bovine meat and fishery industries. Familiar sources include bovine hides, bones, and fish skin, which are often discarded as by-products of food processing^50^. They enhance cell migration, proliferation, and new collagen synthesis, improving wound healing and overall tissue resilience. Indeed, their bioactive properties have stimulated multiple applications in biomaterials, regenerative medicine, and cosmetic formulations to enhance skin elasticity and counteract the effects of aging^51–55^. Additionally, as hydrolyzed fragments, collagen peptides do not retain the native triple-helical structure of collagen. They are therefore already in a denatured state, making the typical denaturation temperatures reported for collagen proteins irrelevant in this context.

This study provides a comprehensive characterization of the developed material’s morphological, structural, mechanical, degradability, biocompatibility, and bioactive properties. Additionally, given that collagen peptides can be obtained from waste, we also investigated whether the properties of the matrix (PCL) may change if the raw material is obtained through a recycling process. Therefore, in conjunction with the novel bioactive 3D-printable composite filament, additional filaments were manufactured and analysed. Their structure has been investigated through morphological and microstructural analyses via scanning electron microscopy, X-ray diffraction, differential scanning calorimetry (DSC), and thermal gravimetric analysis (TGA). To ensure the material’s bioactivity, intrinsic fluorescence analysis was conducted to verify the presence of collagen peptides within the PCL matrix. Lastly, in vitro biocompatibility and degradation analyses have also been performed to assess whether the PCL biocompatibility is preserved after the printing process and to investigate the influence of collagen peptides on the degradation rate of PCL. Finally, examples of 3D-printed scaffolds fabricated using this novel bioactive 3D-printable composite filament are shown to demonstrate that even complex structures can be manufactured. To achieve all these targets, two desktop-based technologies have been used: a filament extrusion system for manufacturing the raw materials and an FFF-based machine for 3D printing them. The intent of using affordable, and especially for the FFF, widely available technologies is driven by the desire to make this material accessible, thereby stimulating the development of new, potential, and practical applications in the healthcare sector.

## Materials and methods

### Materials

Four distinct types of PCL-based materials were investigated, with the first serving as a reference. They are labeled as follows:

1. *PCL Industrial Grade* – a commercially available PCL filament (diameter: 1.75 mm; density: 1.1 g/cm^3^), provided as Facilan™ PCL100 by 3D4Makers (Haarlem, Netherlands).
2. *PCL Lab-Scale* – polymeric bits produced from the *PCL Industrial Grade* filament.
3. *PCL Waste (Lab-Scale)* – polymeric bits obtained from recycled PCL, i.e., by shredding PCL 3D-printed artifacts and scraps resulting from other activities performed in the lab and fabricated using the *PCL Industrial Grade* filament as the raw material.
4. *PCL* + *Collagen peptides* – composite bits generated by solubilizing collagen peptide powder and mixing it with the *PCL Lab-Scale* bits produced starting from the *PCL Industrial Grade* filament. The collagen peptides used in this study are of bovine origin; specifically, they are obtained through the hydrolysis of proteins from meat waste and have been ultrafiltered to obtain an average molecular weight of less than 3 kDa (rich in short and medium-size peptides).

As anticipated, the *PCL Industrial Grade* acts as the reference filament. It is the common denominator among all the studied materials and, therefore, used to study how the manufacturing process influences the material’s morphology and mechanical properties. Indeed, starting from this virgin material, processing and reprocessing steps are implemented to generate the other filaments considered in this study. For example, direct material reprocessing is employed to obtain the *PCL Lab-Scale* filament*,* whereas 3D printing and subsequent reprocessing have resulted in the *PCL Waste (Lab-Scale)* filament*.*

100 g each of *PCL Lab-Scale* and *PCL Waste (Lab-Scale)* material was processed to obtain uniformly sized particles in the form of bits (pellets). These bits were then ball-milled using a Felfil Shredder 750 by Felfil (https://felfil.com) at 33 rpm for 1 h with an applied torque of 36 Nm. Details concerning the preparation and integration of collagen peptides are provided later.

### Fabrication of the *PCL* + *Collagen peptides* bits through solvent-assisted blending

The blending of collagen peptides within the PCL matrix (i.e., the bits) was achieved through the development of an ad hoc multi-step process. The target has been to guarantee complete peptide dissolution and their uniform integration. First, collagen peptides were prepared at a concentration of 1.5% (w/v). In a 95:5 ratio, a solvent mixture of isopropyl alcohol (IPA) and double-distilled water (ddH_2_O) was used for solubilization (Supplementary Fig. S1). The mixture was stirred continuously and heated to 40 °C for 2 h to guarantee the complete dissolution of the collagen peptides. To avoid any abrupt changes in pH, which could trigger premature gelation of the collagen, the ddH_2_O component was added dropwise. Once the solution of collagen peptides was completely solubilized, 100 g of *PCL Lab-Scale* pellets were incorporated into the solution. The resulting blend (labeled as *PCL* + *Collagen peptides*) was stirred for an additional 12 h to ensure the complete evaporation of IPA, effectively allowing the collagen peptides to functionalize the PCL bits. Finally, the composite *PCL* + *Collagen peptides* pellets were transferred to a hot-air oven and dried at 40 °C for 5 h. The final weight ratio of this blend is 80%-20%, i.e., 100 g of PCL and 25 g of collagen peptides. The dried material, now uniformly mixed and blended with collagen peptides, was deemed ready for further processing using the Felfil Evo extruder by Felfil (https://felfil.com). This systematic approach ensured the non-toxic and safe dissolution and integration of the collagen within the PCL matrix, optimizing the material properties for the downstream tests. The selected drying protocol, combined with the temperatures involved in subsequent filament extrusion (130 °C) and 3D printing (165 °C) phases, as later described, will further ensure that no IPA remains in the printed artifacts.

### Extrusion and spooling system for the PCL-based filament fabrication

**Fig. 1:**
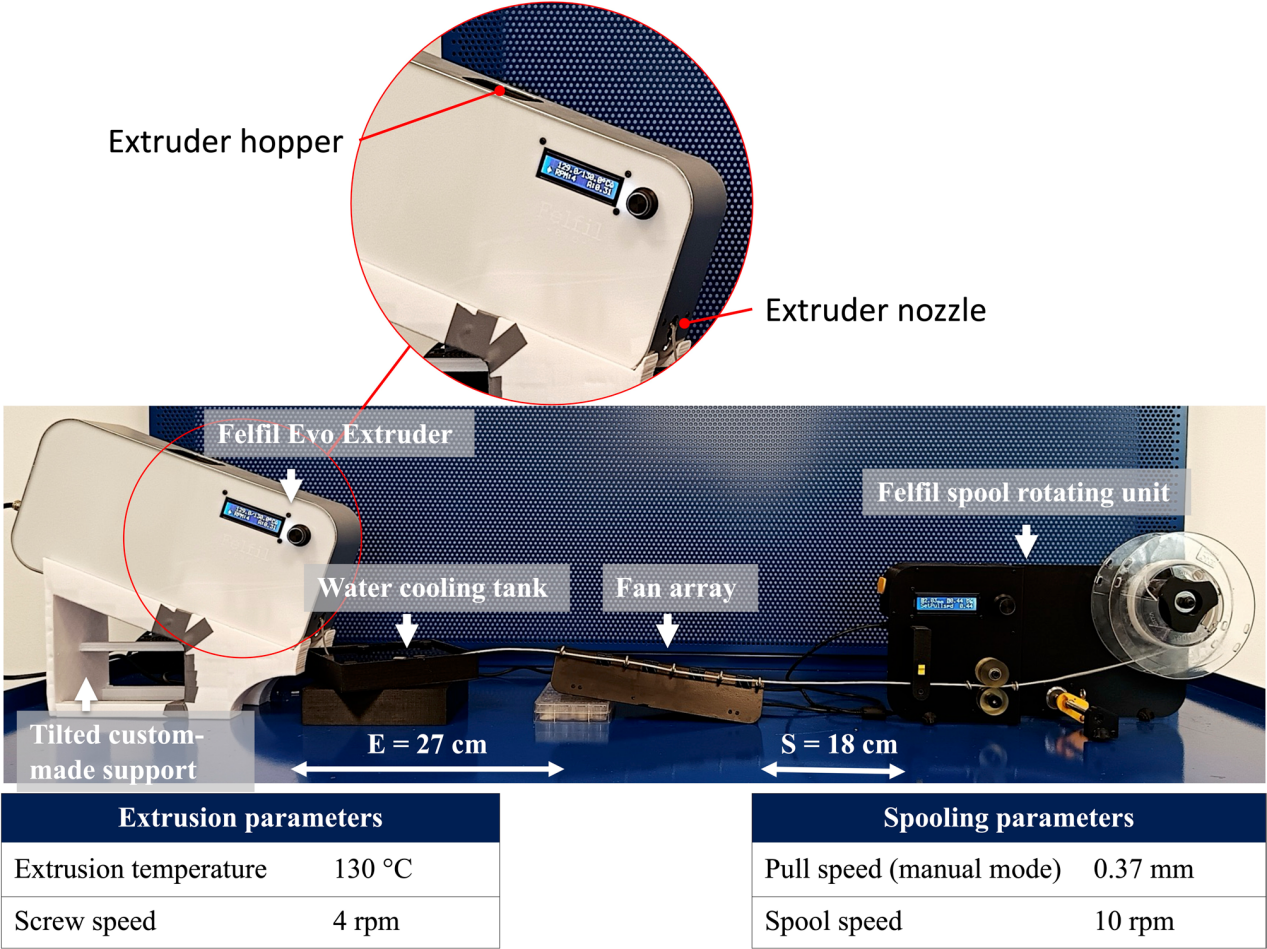
The fine-tuned extrusion system for fabricating PCL-based filaments. The setup includes (from left to right): (1) the Felfil Evo Extruder with its hopper and nozzle and related process parameters; (2) a custom-made support tilted at 20° to enhance polymer detachment and continuous flow; (3) a water cooling tank; (4) a fan array for additional cooling; and (5) the Felfil spool rotating unit and related process parameters. “E” is the distance between the extruder and the fan array, while “S” is the distance between the fan array and the Felfil spool-rotating unit. Those values were selected based on evaluations of the filament quality obtained.

The *PCL Lab-Scale*, the *PCL Waste (Lab-Scale),* and the *PCL* + *Collagen peptides* bits were transformed into filaments using the Felfil Evo extruder. This extrusion system consists of the following components (Fig. 1): 1) the Felfil Evo Extruder—the core unit preheated to 130 °C for initiating the extrusion process; 2) a tilted custom-made support that holds the extruder at an angle of 20°; 3) a water cooling tank and fan that facilitates rapid cooling of the extruded polymer; 4) a fan array; 5) the Felfil spool rotating unit to ensure uniform winding of the filament. The PCL processing parameter supplied by the manufacturer has been listed in Supplementary Table S1. However, optimization of processing parameters was carried out as PCL is a sticky polymer and displays delayed crystallization from the molten state, which causes difficulties in drawing filaments with uniform diameters. Additionally, this stickiness can restrict mass flow through the nozzle, hindering the production of high-quality filaments. To tackle these issues, a custom-made support structure was designed (Supplementary Fig. S2) and utilized to maintain the Felfil Evo extruder in a tilted position (Fig. 1), ensuring a sufficient flow of the PCL bits through the nozzle. Furthermore, a dual cooling system, consisting of a water bath and a fan array, was employed at an optimal distance to direct crystallization in the filaments.

The PCL-based bits were introduced into the extruder’s hopper (Fig. 1). The process parameters were tuned (the extruder is preheated to 130 °C, and the screw speed is set at 4 rpm, Fig. 1) to ensure the polymer extrusion through a 1.75 mm nozzle. At the same time, the spooler was continuously rolling up the filament. To control and optimize filament quality in terms of length, cross-sectional dimensional accuracy, and color, the following parameters were adjusted: extrusion mode (i.e., hard, soft, medium, or manual, according to the extruder’s standard setup), extrusion temperature, screw speed, pull speed, and spool speed. In addition, a novel parameter, the "time of flight" (ToF, i.e., the distance between extrusion and the water-cooling tank, as explained later), was controlled to maintain a consistent filament diameter during the cooling phase.

### Material extrusion (MEX) and process parameters

The FFF Original Prusa XL 3D printer (Prague, Czech Republic) was used to 3D-print all tensile, compression, SEM, and XRD specimens. The PrusaSlicer 2.9 (Prague, Czech Republic) was used to generate the printing instructions. The printer has a maximum building volume of 360 × 360 × 360 mm^3^ and operates at a layer resolution of 0.1 mm, using a nozzle with a diameter of 0.4 mm. The following printing parameters were kept constant for all the tested filaments: nozzle diameter (0.4 mm), infill (100%), layer height (0.2 mm), extruder temperature (165 °C), bed temperature (43 °C), printing speed (28 mm/s), extrusion width (0.46 mm), extrusion multiplier (1.1), retraction distance (0.5 mm), extruder retraction lift (1.5 mm), retraction speed (25 mm/s), wipe distance (3 mm), number of top–bottom solid layers (2), number of perimeter outlines (2). These are the manufacturing parameters used for printing all samples analyzed in this study, including the tensile and compression specimens presented in the following subsection.

### Tensile and compression specimens

Samples were modeled using Autodesk Inventor® software (McInnis Parkway, San Rafael, CA, USA), exported as stereolithography (.stl) format, and then converted into “.gcode” files using the Prusa slicer software. The tensile specimens were designed according to the ASTM D638-14 standard and the dogbone Type IV (Supplementary Fig. S3). Considering the possible FFF-induced anisotropy, the influence of the printing direction (namely, raster angle) was studied. Indeed, 3D-printed FFF artifacts have a raster-based structure that can influence their structural behavior because it determines how the deposition lines, and therefore the material, are oriented with respect to the load (e.g., parallel, perpendicular, or at 45°)^56^. To test this potential influence, the type IV tensile specimens were printed considering four raster angles (0°, 90°, 45°, and 0°-90°, Supplementary Fig. S4). For each experiment, five samples were printed. Prismatic compression specimens (ASTM D695-15), with a nominal cross-sectional area of 12.7 × 12.7 mm^2^ and a height of 25.4 mm, were also designed and printed to evaluate the compressive modulus of the material. For each experiment, three samples were printed.

### Mechanical characterization

Tensile tests were conducted under displacement control using an MTS Synergie 200 universal tensile testing machine equipped with a 1 kN load cell at a crosshead speed of 50 mm/min. Displacement measurements were acquired using an MTS 632.26F-20 extensometer with a gauge length of 25 mm. Quasi-static compression tests were performed using an MTS Alliance RF/150 machine equipped with a 150 kN loading cell at a crosshead speed of 1.3 mm/min, with a maximum crosshead displacement of 12 mm (equivalent to almost half the sample height).

### X-ray diffraction (XRD)

A Rigaku SmartLab SE diffractometer in *θ–θ* configuration was used to obtain the diffraction patterns of the *PCL Industrial Grade*, the *PCL Lab-Scale*, the *PCL Waste (Lab-Scale)*, and the *PCL* + *Collagen peptides* composite. The diffracted beam was detected from 15° to 30° 2*θ*, with a scan rate of 2°/min and a step size of 0.02°, using a D/teX Ultra 250 1D detector. Each sample was tested in triplicate.

### Differential scanning calorimetry (DSC) and thermogravimetric analysis (TGA)

DSC and TGA were performed to evaluate the thermal behavior and degradation profile of all PCL-based samples. The analyses were conducted using the THEMYS ONE by Setaram, equipped with a TG-DSC measuring rod capable of reaching temperatures up to 1600 °C. Each sample (50 mg) was placed in a sealed alumina crucible and subjected to a temperature range of 25 °C to 700 °C, with a heating rate of 10 °C min^-1^ under an argon atmosphere to prevent oxidative degradation. Each sample was tested in triplicate.

### Scanning electron microscopy (SEM)

Morphological characterization of the *PCL Industrial Grade*, *PCL Lab-Scale*, *PCL Waste (Lab-Scale)*, and *PCL* + *Collagen peptides* composite was conducted using a Zeiss EVO 50 scanning electron microscope operating at 30 kV. Both small portions of filament and 3D-printed cubes (5 × 5 × 1 mm^3^) fabricated with that material were analyzed. Before imaging, all samples were sputter-coated with a 4 nm gold layer to enhance conductivity and improve image quality.

### Optical microscopy and intrinsic fluorescence analyses

Optical microscopy and intrinsic fluorescence analyses were employed to investigate the blending of collagen peptides with the *PCL Lab-Scale* matrix. 3D-printed samples were used. First, the structural characteristics of *PCL Lab-Scale* and *PCL* + *Collagen peptides* specimens were compared using a Zeiss Axio Vert A1 optical microscope. Then, intrinsic fluorescence analysis was conducted to confirm the presence of collagen peptides. For this purpose, the same specimens as before—along with pure collagen peptide powder (the positive control) and *PCL Industrial Grade* (the negative control)—were analyzed. Samples were excited at 266 nm^57^, and the fluorescence emissions were recorded using a BioTek Synergy H1 Multimode Microplate Reader (Agilent). Each sample was tested in triplicate.

### In vitro biocompatibility

To evaluate the biocompatibility of PCL-based materials, it is necessary to exclude any adverse effects on human fibroblast viability. To this aim, MTT assays were performed in contact with fibroblasts using 3D-printed cylindrical samples (diameter of 6.5 mm and height of 2 mm) manufactured using the *PCL Industrial Grade*, *PCL Lab-Scale*, and *PCL* + *Collagen peptides* materials. The cells used in this study were commercially available hTERT-immortalized human skin fibroblasts (BJ-5ta; ATCC® CRL-4001™), purchased from LGC Standards (Milan, Italy). Therefore, no ethical approval was required. The MTT assay measures cellular metabolic activity as an indicator of cell viability, proliferation, and cytotoxicity. Specifically, 2 × 10^4^ BJ-5ta cells/well were seeded in 96-multiwell plates and, after 24 h, treated with the 3D-printed samples and/or vehicle (H_2_O) in complete growth medium for 48 h at 37 °C in a 5% CO_2_ atmosphere. Then, the culture media were discarded, and the 3-(4,5-dimethylthiazol-2-yl)-2,5-diphenyltetrazolium bromide MTT solution was added (5 mg/mL of solution in phosphate-buffered saline). The plates were then incubated for 2 h. After that, the MTT solution and the samples were removed, and 100 μL of lysis buffer was added per well. The plates were shaken for 10 min, and the absorbance was measured at 575 nm using a Synergy H1 fluorescence plate reader (Biotek, Bad Friedrichshall, Germany).

### In vitro degradation

The degradation behavior of PCL-based materials was evaluated by measuring the weight loss of filaments under physiological conditions. Specifically, filaments of *PCL Industrial Grade*, *PCL Lab-Scale*, and *PCL* + *Collagen peptides* were immersed in phosphate-buffered saline (PBS, 10 mg/mL, pH 7.4; Sigma-Aldrich) and incubated at 37 °C to simulate in vivo conditions. At predetermined time intervals, the samples were removed from the PBS and gently dried before weighing. The degradation rate was calculated as the percentage of weight loss relative to the initial mass, using the following formula:

1 $${\mathrm{Degradation}}\;{\mathrm{rate}}\left( \% \right) = \left[ {\left( {{\mathrm{W}}_{0} {-}{\mathrm{W}}_{{\mathrm{t}}} } \right)/{\mathrm{W}}_{0} } \right] \times {1}00$$

where W_0_ is the initial dry weight of the filament, and W_t_ is the dry weight at the specified time point.

### Design and printing of triply periodic minimal surface (TPMS)–based porous scaffolds

3D models of the Gyroid, Diamond, Honeycomb Gyroid, Split-P, and Lidinoid porous scaffolds were generated using the Functional Lattice Package (FLatt Pack) software^58^. For all these TPMS-based structures, the matrix configuration (also referred to as “sheet”^58^) was selected. Each structure was designed as a linear repetition of 2 × 2 × 1 cells in each Cartesian direction with a 6 mm cell size, and a volume fraction (ρ) of 0.3. The models were exported as “. stl” files and sliced using the PrusaSlicer 2.9 (Prague, Czech Republic). To manufacture these structures, the tuning of the printing parameters has been performed as follows: the extrusion width was decreased from 0.46 mm to 0.42 mm; the printing speed was set to 10 mm/s to ensure more accurate material deposition on each layer; the layer height was reduced to 0.15 mm to enhance printing resolution, and a retraction distance of 0.8 mm was applied between layers to prevent stringing. The *PCL* + *Collagen peptides* material has been used to fabricate them.

### Statistical analysis

Statistical analysis was carried out by one-way ANOVA (GraphPad Prism 9) followed by Tukey’s multiple comparisons tests. Values were expressed as means ± SEM; *p*-values < 0.05 were significant.

## Results and discussion

### Filament fabrication

As shown in Fig. 1, the distances between the extruder and the fan array (E) and the one between the fan array and the spooling unit (S) were set to 27 cm and 18 cm, respectively, to prevent filament sagging and ensure adequate pulling action during material extrusion. This setup allowed a consistent PCL flow from the extruder to the spooling unit. Additionally, since PCL is a low-melting polymer, the standard air-cooling equipment available was insufficient alone to rapidly cool the extruded material, which, being still in a semi-molten state, adhered to the fan array’s eyebolts. To address this issue, a room-temperature water-cooling system (Fig. 1) was installed directly under the extruder, enabling the material to solidify immediately upon contact with the water surface, thereby enhancing the filament’s structural integrity.

Preliminary tests indicated that PCL’s high surface energy^59^, causes it to adhere to the extruder’s metallic nozzle (Fig. 1). To tackle this challenge, the extruder was tilted using an ad-hoc support structure. The target was to promote rapid material detachment and ensure continuous extrusion (Supplementary Fig. S2). The support was designed to implement a 20° tilting angle (α). Further design features considered were: 1) a frontal-bottom opening to accommodate the water tank, ensuring water contact well away from the tank edges; 2) a central opening to make it easier for the filament to fall directly into the water tank; 3) perimeter walls to keep the extruder in place (Supplementary Fig. S2). Implementation of this support improved the uniformity of the PCL flow at the nozzle and enhanced the distribution of polymer fragments into the extrusion screw’s threaded section, facilitating more efficient pellet collection. Furthermore, the time-of-flight (ToF) of the polymer between the extruder and the water tank was found to significantly influence the diameter of the filament. Since the ToF depends on the distance between the nozzle and the water surface, this parameter was tuned by adjusting the geometrical variables α and *z* (Supplementary Fig. S2). With the ramp’s geometrical feature (α) held constant, the water surface height in the tank was increased to 65 mm, resulting in an optimized *z* value of 43.21 mm.

**Fig. 2:**
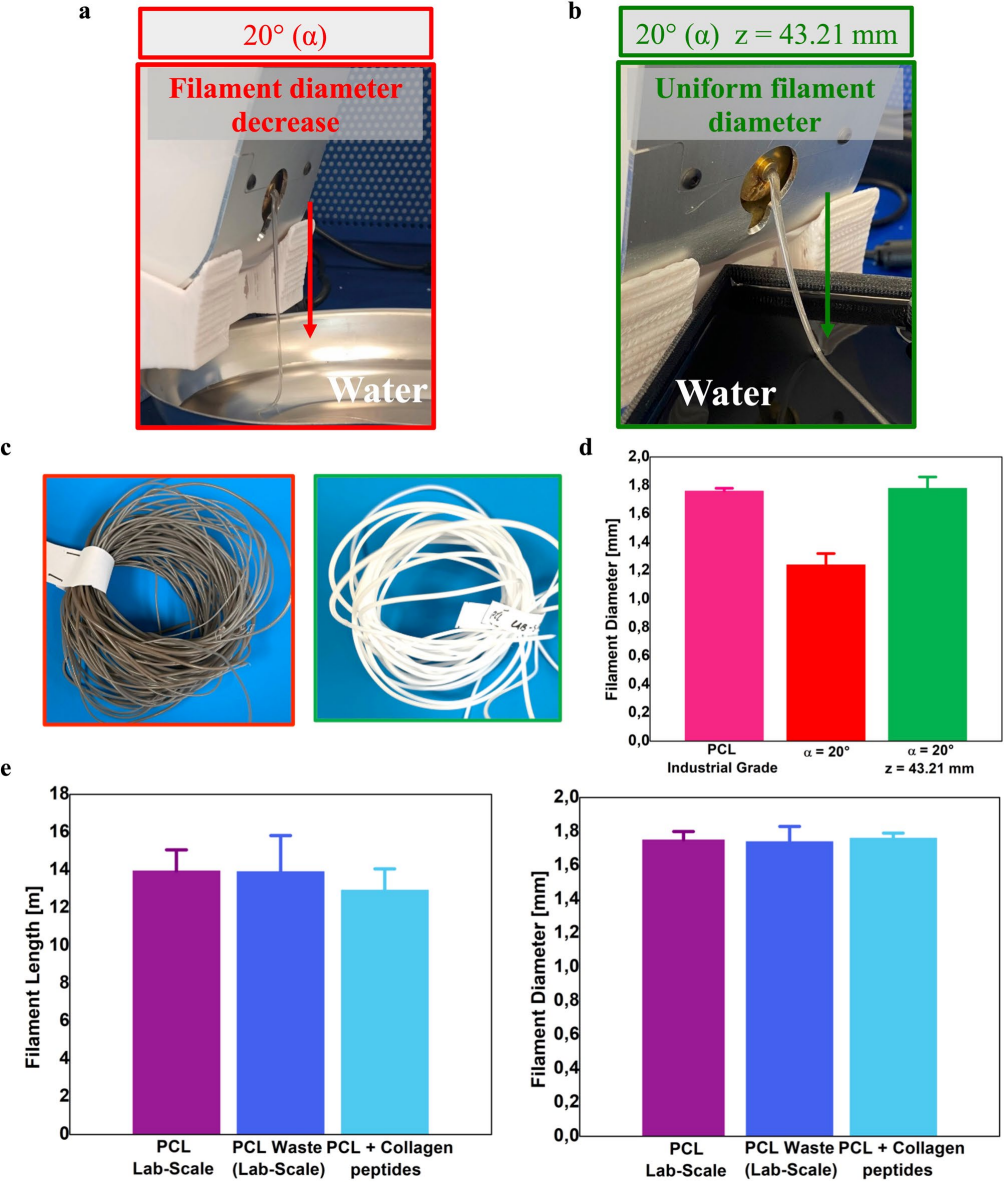
Overview of the filament extrusion issue and main results. (**a**) The filament extruded without the time-of-flight (ToF) optimization, resulting in a decrease in the filament diameter. (**b**) Filament extruded with the optimized ToF, resulting in a constant filament diameter. (**c**) Comparison of extruded defective filament (black) versus a high-quality filament (white, ~ 1.75 mm diameter). (**d**) Comparison of PCL filament diameters: the *PCL Industrial Grade* filament (in pink) and the extruded *PCL Lab-Scale* with (in green) and without (in red) the ToF integration. (**e**) Distribution of filament lengths and corresponding diameters achieved with the optimized extrusion process.

Figure 2a–c compares two configurations: one using only a 20° tilting angle and another combining the same tilt with optimized ToF (43.21 mm). Without the ToF adjustment, the filament diameter was inconsistent, averaging 1.24 ± 0.08 mm (Fig. 2d) and too narrow for adequate feeding through the 3D printer’s gears. Conversely, integrating the 20° tilt with the ToF optimization prevented sticking and maintained a consistent filament diameter from the nozzle to the cooling system. This approach yielded an average diameter of 1.76 ± 0.08 mm, closely aligning with industrial-grade PCL filaments’ 1.75 mm nominal diameter (Fig. 2d). Without these optimizations, several issues were observed: the PCL tended to adhere to the metal extruder nozzle of the Felfil system, the filament exhibited significant diameter variability (1.02 ± 0.13 mm), rendering it unsuitable for 3D printing (Supplementary Fig. S5), and the overall filament yield was reduced, resulting in a final length of only 1.42 m from the same initial mass of material.

Following the implementation of the optimized configurations for PCL-based filaments, the extrusion parameters were fine-tuned for all PCL-based materials under investigation. Initial settings were derived from Felfil manufacturer guidelines (Supplementary Table S1) and supported by previous studies^60,61^.

The impact of extrusion temperature was first evaluated. Increasing the temperature from 95 °C to 130 °C resulted in improved process consistency, enhanced control over the filament diameter, and increased process repeatability. The screw speed was set to 4 rpm. Trials with higher speeds (e.g., 8 rpm) led to material accumulation before the water tank and fan array, complicating the process control. Lower speeds (e.g., 3 rpm) could not guarantee continuous extrusion.

Furthermore, the process demonstrated sensitivity to the pull speed. This parameter affects the material flow and the filament diameter. Since the “soft extrusion mode” setting does not allow controlling the pull speed, the extruder was switched to the “manual mode”; the pull speed was then set to 0.37 m/min. To maintain a uniform process flow and prevent failure, the spool speed was synchronized with the pull speed and set to 10 rpm. An overview of the extrusion parameters is provided in Table 1.

**Table 1:** Optimized extrusion parameters, filament length, and average diameters for all PCL-based materials analyzed in this study. The mass parameter indicates the grams of bits used.

Material	Mass (g)	Temperature (°C)	Screw speed (rpm)	Pull speed (m/min)	Spool speed (rpm)	Filament length (m)	Avg. filament diameter (mm)
PCL Lab-Scale	40	130	4	0.37	10	13.95 ± 1.12	1.75 ± 0.05
PCL Waste (Lab-Scale)	40	130	4	0.37	10	13.91 ± 1.91	1.74 ± 0.09
PCL + Collagen peptides	40	130	4	0.37	10	12.93 ± 1.13	1.76 ± 0.03

For each PCL-based material, the extruded filament diameters matched those of the *PCL Industrial Grade*. Also, filament lengths were consistent with the selected pellet mass (Table 1). Applying the same process parameters across all PCL-based pellets led to uniform filament diameters and lengths (Fig. 2e, Table 1). These results confirm the repeatability and scalability (in terms of materials) of the optimized lab-scale extrusion process.

### Morphological characterization

**Fig. 3:**
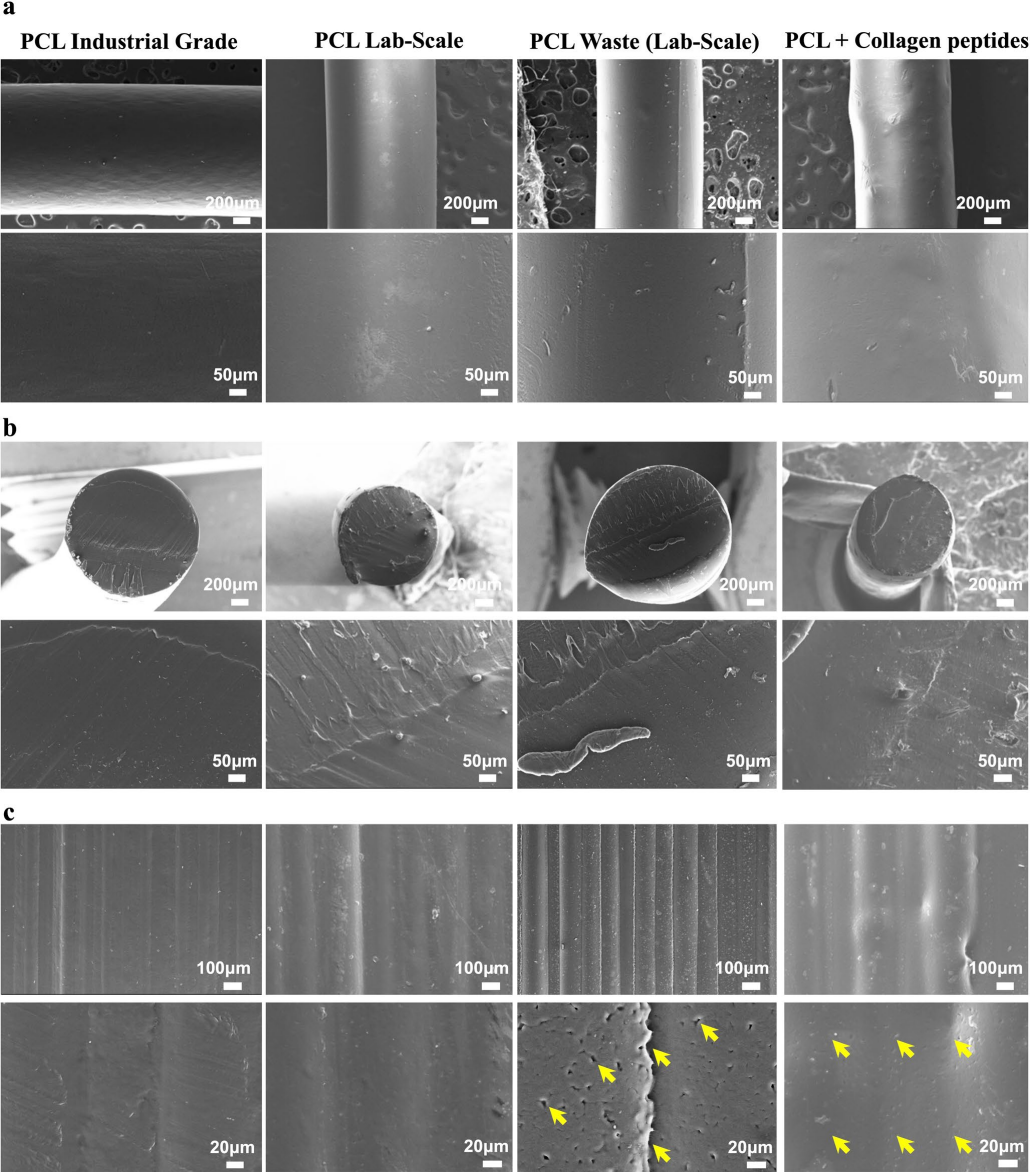
SEM images (from left to right) of the *PCL Industrial Grade*, *PCL Lab-Scale*, *PCL Waste (Lab-Scale)*, and *PCL* + *Collagen peptides* materials. (**a**) SEM images of the external surfaces of the filaments. The industrial and lab-scale filaments exhibit smooth, high-quality surfaces; the composite filament shows irregularities due to the presence of collagen peptides. (**b**) Cross-sectional SEM images of the filaments. They all exhibit uniform diameters. We can also observe minimal porosity in both lab-scale and recycled (i.e., *PCL Waste*) samples, as well as the successful embedding of collagen peptides within the composite matrix (i.e., the *PCL* + *Collagen peptides*). (**c**) SEM images acquired from the 3D-printed specimens to study the interlayer adhesion. The *PCL Industrial Grade* and *PCL Lab-Scale* samples demonstrate uniform and robust bonding. The *PCL Waste (Lab-Scale)* specimens reveal pores and gaps between layers (the arrows highlight their presence). The *PCL* + *Collagen peptides* samples exhibit fused interlayers, indicating a modified thermal behavior upon integration of collagen peptides (the arrows highlight these fused interlayers).

Figure 3a presents the SEM images of the external surface morphology for the *PCL Industrial Grade*, *PCL Lab-Scale*, *PCL Waste (Lab-Scale)*, and *PCL* + *Collagen peptides* filaments. The PCL *Lab-scale* and the *PCL Waste (Lab-Scale)* filaments show a high-quality surface appearance (like the *PCL Industrial Grade*). However, some unprocessed polymer residues from the shredded bits are visible. In contrast, the *PCL* + *Collagen peptides* filament shows a surface with irregularities, likely due to the presence of the collagen peptides. These asperities are advantageous for biomedical applications, as they may promote enhanced cell adhesion, proliferation, and growth on 3D-printed scaffolding^62^.

Figure 3b presents the cross-sectional morphology of these filaments. The SEM images confirm that the extruded filaments have a consistent diameter comparable to that of the industrial-grade. Minimal porosity is observed in the *PCL Lab-scale* and *PCL Waste (Lab-Scale)* filaments due to the mechanical shredding process used to mill the material before extrusion. In addition, highly magnified views reveal the presence of fringes, indicating that residual stresses were induced during the thermomechanical processing.

All filaments demonstrated properties suitable for FFF 3D printing (Fig. 3c). An evaluation of the interlayer adhesion in 3D-printed samples shows that the *Industrial* and *Lab-scale* PCL filaments exhibit uniform and robust bonding. However, the *PCL Waste (Lab-Scale)* samples reveal pores and gaps between the layers; this effect is due to the material being obtained from bits generated from 3D-printed scraps or artifacts. Additionally, the infill in the *PCL* + *Collagen peptides* samples shows fused interlayers, indicating that the incorporation of collagen alters the thermal behavior of the filament during printing.

Overall, SEM analysis reveals some differences, especially for the *PCL Waste (Lab-Scale)* material, in terms of morphology, compared to the *PCL Industrial Grade*, which serves as the reference. However, despite these differences, the 3D printing process was successfully concluded for all materials, demonstrating that all the filaments can be 3D-printed, albeit with varying quality. Therefore, the reproducibility and robustness of the optimized extrusion process are confirmed, as well as the microstructural impacts of recycling and collagen incorporation on the PCL matrix.

### Structural characterization

**Fig. 4:**
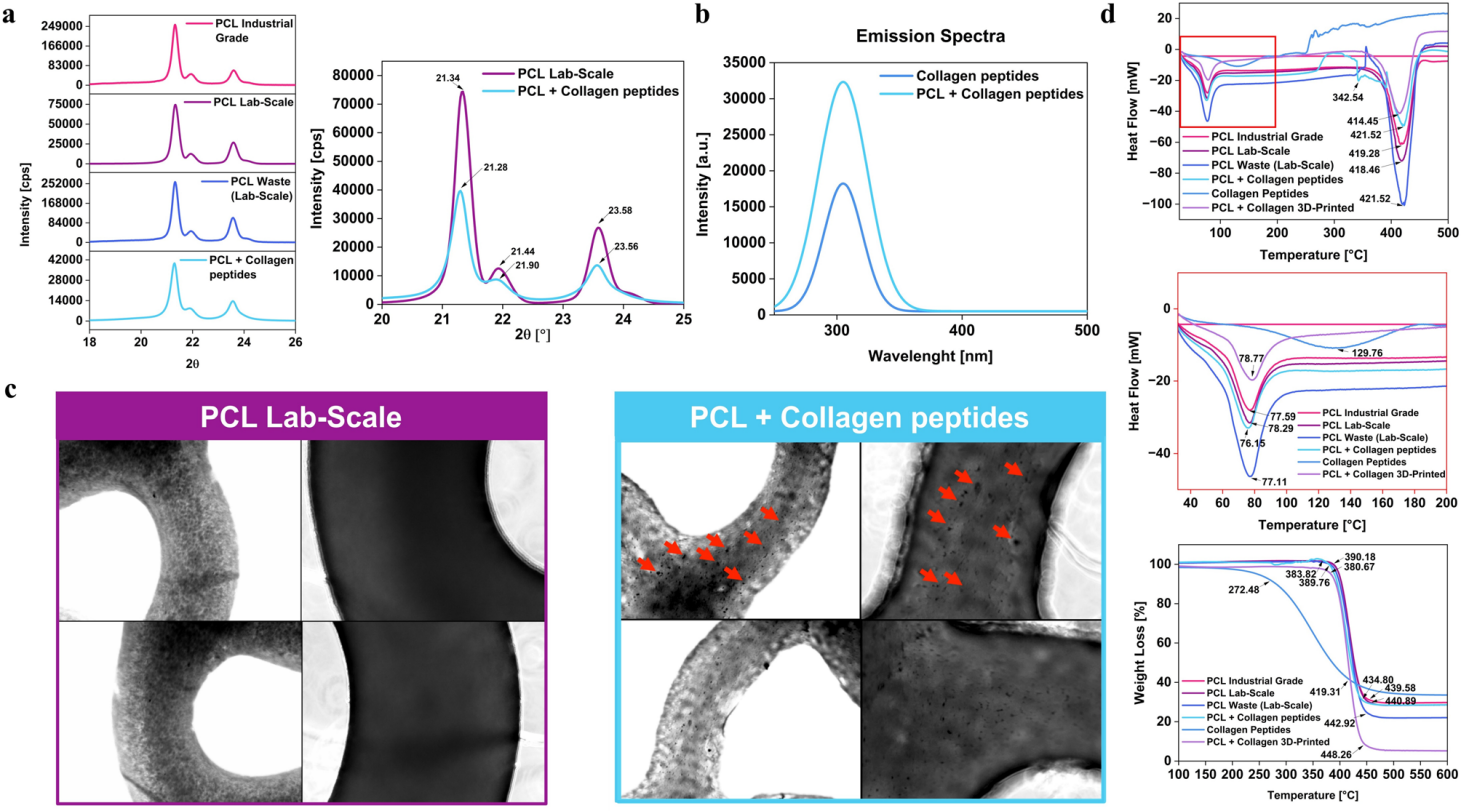
Structural and thermal characterization of PCL-based filaments, 3D-printed artifacts, and collagen peptide powder. (**a**) X-ray diffraction patterns of the *PCL Industrial Grade*, *PCL Lab-Scale*, *PCL Waste (Lab-Scale)*, *PCL* + *Collagen peptides*, with a focus on the characteristic reflections and shifts resulting from the presence of collagen peptides. (**b**) Intrinsic fluorescence spectra of pure collagen peptides and *PCL* + *Collagen peptides*; emission peaks in the UV–vis region due to tyrosine residues of collagen peptides are visible. (**c**) The presence of collagen peptides within the PCL matrix after 3D printing (image acquired from 3D-printed samples) is confirmed by optical microscopy; (**d**) DSC and TGA analyses to monitor the glass transition, degradation behavior, and two-stage weight loss trends of the tested filaments and 3D-printed *PCL* + *Collagen peptides* materials.

The diffraction patterns (Fig. 4a) revealed corresponding reflections for all PCL filaments and the presence of the collagen peptides in the *PCL-Collagen peptides* composite sample. The *PCL Industrial Grade*, *PCL Lab-Scale*, *PCL Waste (Lab-Scale)*, and *PCL* + *Collagen peptides* filaments exhibited two characteristic peaks between Bragg’s angles 2*θ* = 21 and 24°, attributed to the 110, 200 planes with a typical orthorhombic unit cell structure of PCL and its semi-crystalline nature^63^. The intense and similar peaks for all three grades of PCL samples suggest that the crystallinity is preserved in the recycled samples. Furthermore, the diffraction peaks of PCL were evident in the *PCL* + *Collagen peptides* composite; a decline and minor shift (< 0.5°) could also be observed, although the amorphous collagen peak, typically occurring at 2*θ* = 24.52°, was absent, which can be attributed to peptide overlapping and their minimal content^64,65^. This disruption in the intensities of the PCL peak indicates a reduction in the degree of crystallinity due to amorphous scattering resulting from disoriented collagen peptide entities, hence confirming its presence; however, due to the lower content of collagen, its corresponding peaks are inconspicuous^66^. Moreover, the decline in crystallinity can be attributed to the interaction between the collagen and the amorphous phase of PCL, resulting in alterations to the PCL structure^67^.

Subsequently, intrinsic fluorescence data also supported the presence of collagen peptides in the composite (Fig. 4b)^68^. Both fluorescence peaks appear in the UV–vis region (300–350 nm), consistent with the emission characteristics of collagen peptides. This fluorescence is typically attributed to tyrosine residues, which are excited at approximately 280 nm^69^. The intensity of the emission spectrum for pure collagen is higher than the spectra exhibited by *PCL* + *Collagen peptides*, which is associated with the presence of collagen peptides at lower concentrations^70^. Moreover, it implies that collagen peptides were solely responsible for fluorescence, given that PCL lacks inherent fluorescence characteristics^71,72^. The presence of collagen peptides in the PCL matrix and after the 3D-printing phase was further confirmed by optical microscopy images (indicated by the arrow in Fig. 4c). A comparable fluorescence behavior was observed even when the *PCL* + *Collagen peptides* composite was embedded in an aqueous environment for 96 h (Supplementary Fig. S6). In this case as well, the presence of collagen peptides within the PCL matrix (after removal of the composite from the aqueous environment) was further confirmed through optical microscopy images (Supplementary Fig. S6). This suggests that collagen peptides remained at or near the surface of the filaments, thereby preserving their bioactive role in promoting cell adhesion.

Lastly, differential scanning calorimetry (DSC) and thermogravimetric analysis (TGA) were performed to evaluate the thermal behavior of all the tested materials (Fig. 4d). DSC results revealed the occurrence of initial endothermic peaks at 77.59 °C (for the *PCL Industrial Grade*), 78.29 °C (for the *PCL Lab-Scale*), 77.11 °C (for the *PCL Waste Lab-Scale*), and 76.16 °C (for the *PCL* + *Collagen peptides*), which is around the glass transition temperature (T_g_) of PCL, being the principal constituent in all the samples^73^. Moreover, a second endothermic peak was also observed for the *PCL* + *Collagen peptides* sample, typically attributed to the degradation of the collagen polypeptide chain and the evaporation of strongly bonded water^74^. The third endothermic peak at approximately 419 °C could be related to the thermal degradation temperature of PCL. Furthermore, the presence of collagen has disturbed the crystallinity of the *PCL* + *Collagen peptides* composite, as also observed in the X-ray analysis. Instead, the collagen peptide powder showed the endothermic peak at 129.76 °C. The TGA curves (Fig. 4d) show a two-stage weight loss. The *PCL* + *Collagen peptides* dipped around 384 °C, with a residual weight of ~ 28% at 435 °C. The *PCL Industrial Grade, PCL Lab-Scale,* and *PCL Waste (Lab-Scale)* are similar in their initial stages. However, a slight increase is observed in the second stage for the *PCL Waste (Lab-Scale)* at ~ 442 °C and with a residual weight of at least 22%. Finally, the thermal analysis of the 3D-printed *PCL* + *Collagen peptides* composite revealed a thermal behavior consistent with that of the non-printed *PCL* + *Collagen peptides* formulation, as expected. Both DSC and TGA data confirmed the preservation of key thermal transitions, including the initial endothermic peak near the Tg of PCL (~ 76 °C) and a secondary peak attributed to the degradation of the collagen peptides. The third peak near 419 °C, indicative of the PCL thermal degradation, was also retained in the printed composite. Similarly, the two-step weight loss pattern observed in the TGA analysis matched that of the original blend, indicating that the 3D printing process did not alter the material’s thermal characteristics.

Overall, the structural characterization of PCL-based filaments has shown how recycling and incorporating collagen peptides affect the PCL matrix. X-ray diffraction confirmed that recycled PCL has the same crystallinity as the virgin PCL and that the successful blending of collagen peptides in the matrix is achieved despite their low content. The fluorescence data confirmed the presence of collagen peptides, with emissions consistent with tyrosine residues. The recycled PCL filament retains the same thermal properties as the industrial and lab-scale ones, with minimal variations in glass transition and degradation temperatures. Additionally, the inclusion of collagen peptides has a minor influence on crystallinity, as evidenced by XRD and DSC data.

Hence, these findings demonstrate that the PCL’s structural and thermal properties are preserved in both lab-scale and recycled PCL conditions. Additionally, the incorporation of collagen peptides introduces bioactive potential with minor effects on the PCL crystallinity and thermal behavior.

### In vitro biocompatibility and degradation

**Fig. 5:**
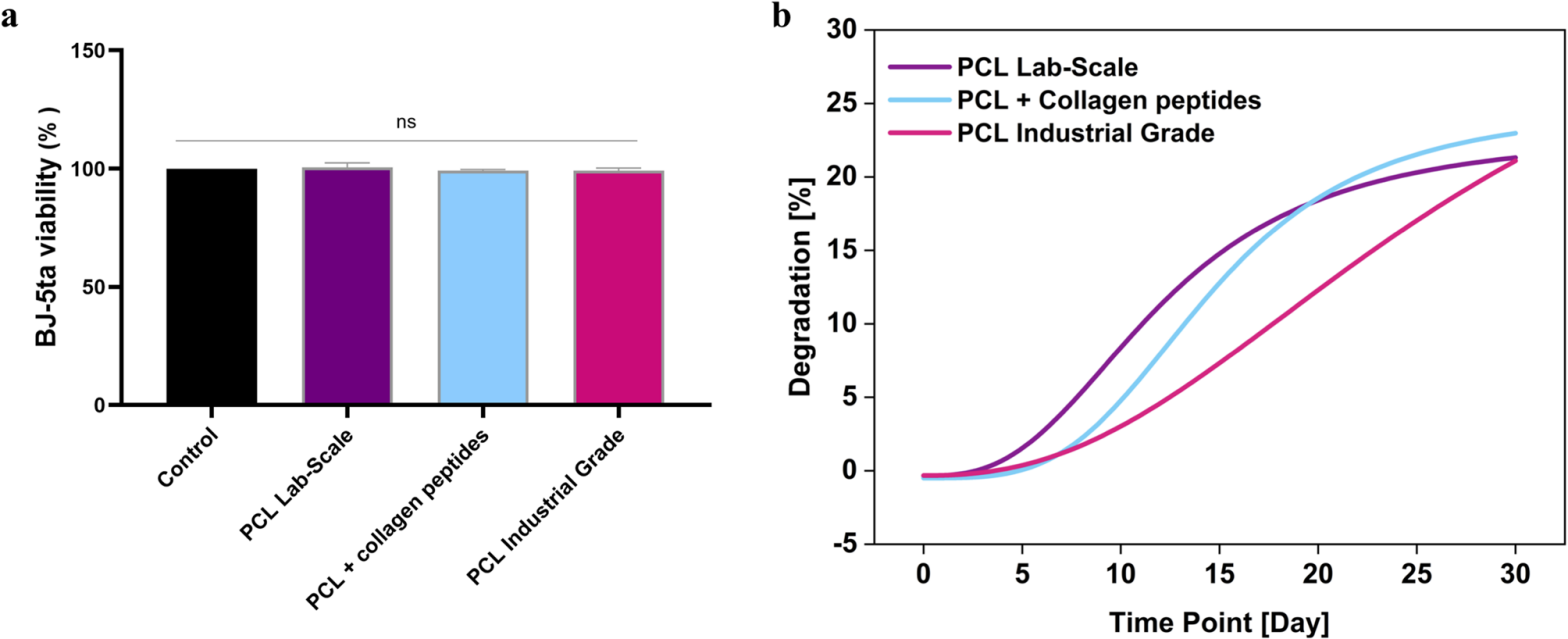
In vitro MTT assay and degradation profiles of PCL-based filaments under simulated physiological conditions. (**a**) MTT assay of *PCL Lab-Scale*, *PCL* + *Collagen peptides*, and *PCL Industrial Grade* on BJ-5ta cells. The bars represent the mean ± s.d. of three independent experiments performed in triplicate and statistically analyzed by One-way ANOVA followed by Tukey’s post-hoc test. Ctrl: control, ns: not significant. (**b**) The graph illustrates the weight loss over time for the *PCL Industrial Grade*, *PCL Lab-Scale*, and *PCL* + *Collagen peptides* materials. The *PCL Lab-Scale* exhibited the earliest onset of degradation, reaching 10% by Day 6, while the *PCL Industrial Grade* showed a delayed and gradual profile, reaching 10% by Day 14 and 21% by Day 30. The *PCL* + *Collagen peptides* demonstrated the most accelerated degradation, with weight loss rising sharply from 11 to 22% between Days 12 and 20.

Concerning biocompatibility, as shown in Fig. 5a, no significant changes in fibroblast cell viability were observed across the 3D-printed samples, compared to the control (untreated) group. Moreover, the degradation profiles (Fig. 5b) of PCL-based filaments under simulated physiological conditions revealed significant differences among the three tested formulations (*PCL Industrial Grade*, *PCL Lab-Scale*, and *PCL* + *Collagen peptides*), highlighting the influence of material origin and composite formulation on hydrolytic stability.

The *PCL Lab-Scale* exhibited the earliest onset of degradation, with weight loss reaching approximately 10% by Day 6. This faster degradation may be attributed to the lab-scale extrusion process, which may affect degradation kinetics. Conversely, the *PCL Industrial Grade* demonstrated a more delayed degradation profile, with measurable weight loss initiating only after Day 8 and reaching 10% by Day 14. Its gradual degradation progression, with a 21% weight loss by Day 30, is consistent with the known stability of medical-grade PCLs, which are often optimized for slower resorption. The *PCL* + *Collagen peptides* composite showed the most pronounced degradation rate, particularly between Days 12 and 20, during which the weight loss increased sharply from 11 to 22%. This behavior is likely driven by the hydrophilic and bioactive nature of collagen, which can enhance water uptake and promote more extensive hydrolysis of the surrounding PCL matrix. Such accelerated degradation is advantageous for applications where faster bio-resorption and tissue integration are desired, including soft tissue scaffolds and wound healing devices.

By the end of the testing period, all three formulations exhibited similar overall degradation levels, with no statistically significant difference detected, despite differences in degradation kinetics and onset times. This convergence in final weight loss suggests that while the degradation rate can be modulated through formulation and processing, the ultimate resorption potential of PCL-based materials remains comparable under the studied conditions.

The results emphasize the tunability of the PCL degradation rate through both material selection and formulation. While industrial-grade PCL offers long-term stability, lab-scale and collagen-integrated variants present promising options for scenarios demanding faster degradation profiles.

### Mechanical characterization

**Fig. 6:**
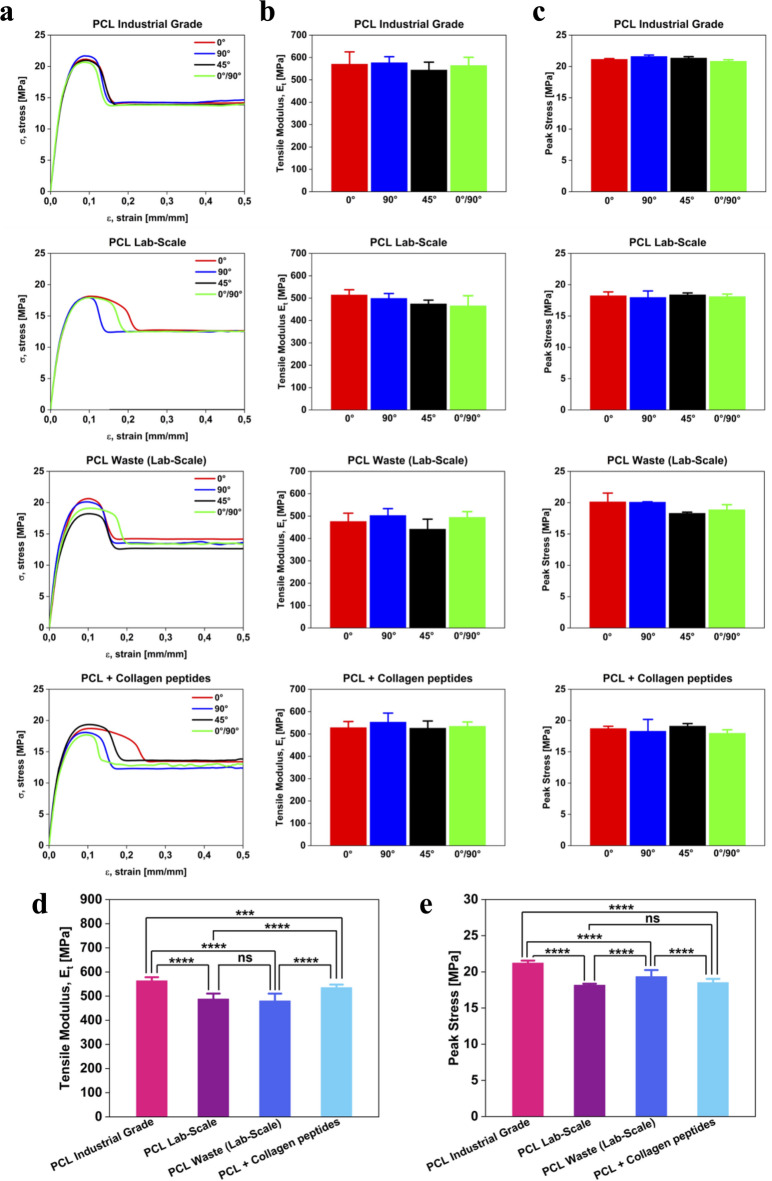
Mechanical characterization of PCL-based specimens. (**a**) Average tensile stress–strain curves for the *PCL Industrial Grade*, *PCL Lab-Scale*, *PCL Waste (Lab-Scale)*, and *PCL* + *Collagen peptides*; (**b**) Tensile moduli for each material and raster angles (0°, 90°, 45°, and 0°-90°). (**c**) Peak stress values for each material; (**d** and **e**) Statistical analyses of the tensile modulus (**d**) and peak stress (**e**), with highlighted significant differences (^*^p-values < 0.05) among the samples.

The tensile tests (Fig. 6) showed no effect of the raster angles (0°, 90°, 45°, and 0°-90°) on the mechanical properties. All samples exhibited similar isotropic-like mechanical behavior along all printing directions (Fig. 6a–c). The ANOVA analysis further corroborated this observation. The tensile moduli analysis revealed significant differences among the investigated materials (Fig. 6d). The *PCL Industrial Grade* exhibited the highest modulus, reaching 564 ± 14 MPa. The *PCL Lab-Scale* one decreased by 76 ± 36 MPa (i.e., 488 ± 22 MPa). This decrease could be attributed to the shredding and lab-scale extrusion processes. The *PCL Waste (Lab-Scale)* exhibited a tensile modulus of 479 ± 27 MPa, slightly lower than that of the *PCL Lab-Scale* but comparable. These findings demonstrate that the recycling process does not significantly compromise the tensile properties of PCL. The *PCL* + *Collagen peptides* material reached 536 ± 12 MPa, representing the highest value among the “lab-scale” ones. Collagen peptides, therefore, act as a reinforcing mechanism, i.e., as a filler, enhancing the mechanical stiffness of the base *PCL Lab-Scale* material. This effect was observed in specimens printed along the 90° raster angle, indicating a strong interaction between the PCL matrix and the collagen peptides. This result confirms the effectiveness of the developed solubilization protocol. At the molecular level, collagen peptides contain amine (–NH_2_) and hydroxyl (–OH) functional groups, which can form hydrogen bonds with the carbonyl (C=O) groups of the PCL^75^. This intermolecular bonding could promote the adhesion between the peptides and the PCL matrix, facilitating stress transfer and increasing the material’s stiffness. These findings highlight the potential of the *PCL* + *Collagen peptides* composite in applications requiring bioactive and mechanically robust materials. For completeness, the mean values of the tensile modulus for each sample with different raster angles and printing temperatures are summarized in the Supplementary Table S2. Peak stress analysis was performed for each material (Fig. 6c). Again, the *PCL Industrial Grade* displayed the highest peak stress value of 21.22 ± 0.33 MPa (Fig. 6e). Instead, the *PCL Lab-Scale*, the *PCL Waste (Lab-Scale)*, and the *PCL* + *Collagen peptides* reached the peak stresses of 18.18 ± 0.18 MPa, 19.35 ± 0.18 MPa, and 18.53 ± 0.50 MPa, respectively.

**Fig. 7:**
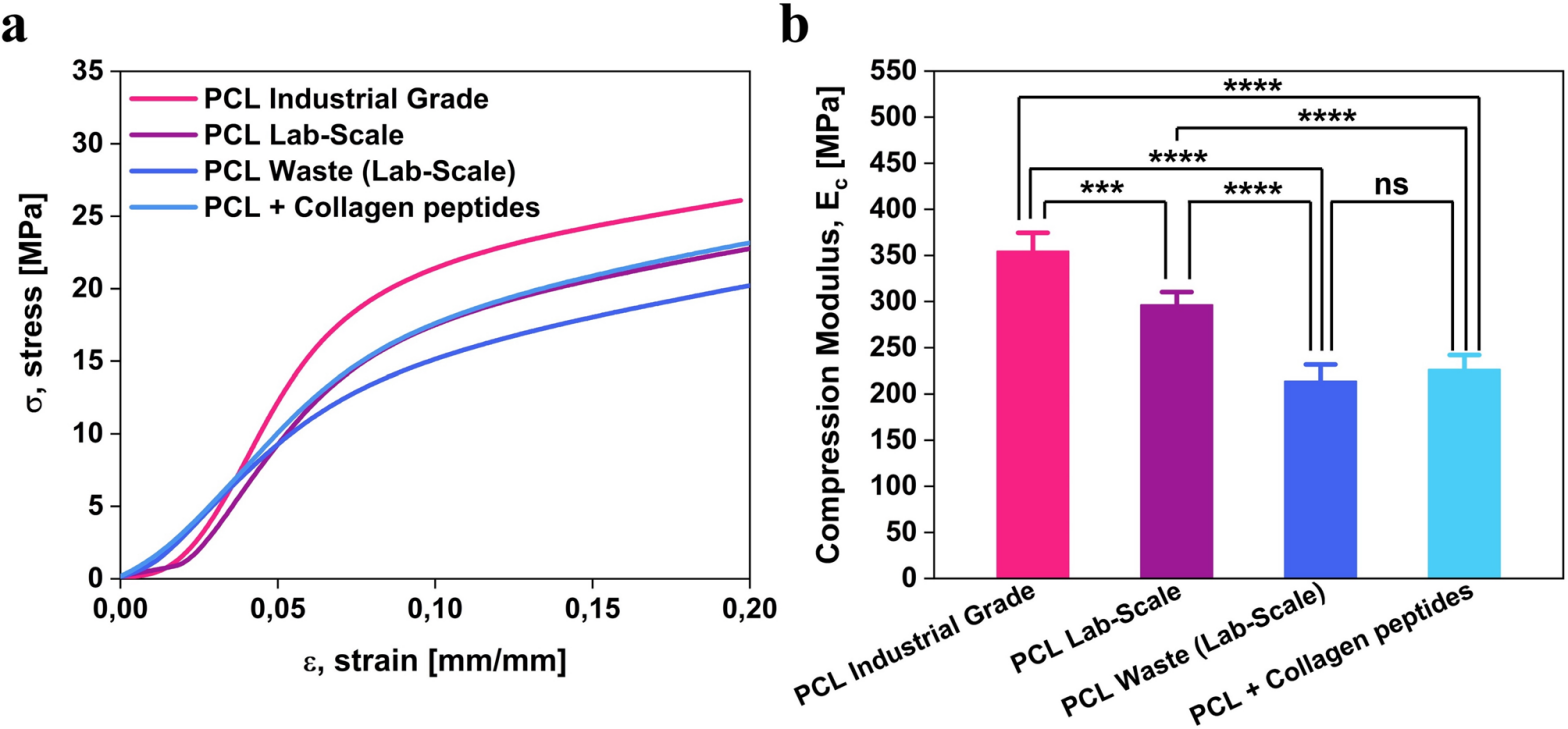
Compressive mechanical performance of PCL-based materials. (**a**) Average stress–strain curves for the *PCL Industrial Grade*, *PCL Lab-Scale*, *PCL Waste (Lab-Scale)*, and *PCL* + *Collagen peptides* materials. (**b**) Statistical comparison of compressive modulus values, demonstrating the effects of recycling and collagen incorporation on compressive strength. ^*^p-values < 0.05 were considered significant.

Finally, compression tests revealed significant differences among the analyzed materials (Fig. 7a–b). The *PCL Industrial Grade* exhibited the highest compression modulus with a mean value of 355 ± 20 MPa. The *PCL Lab-Scale* has a modulus (i.e., 296 ± 14 MPa) that is lower than that of the *PCL Industrial Grade*. This outcome supports the hypothesis that the lab-scale extrusion process induces morphological alterations (as shown in the SEM analysis, Fig. 3), which in turn reduce mechanical properties. The *PCL Waste (Lab-Scale)* exhibited the lowest modulus among all PCL-based materials (214 ± 18 MPa), probably because the recycled feedstock has been exposed to environmental stressors, such as UV radiation, thermal cycling, and moisture. Lastly, the *PCL* + *Collagen peptides* reached a compressive modulus of 227 ± 15 MPa, comparable to that of *PCL Waste (Lab-Scale)*. This result fits with the general trend observed in polymer composites, where a secondary phase (in our case, the collagen peptides) can decrease the compressive performance of the matrix.

This mechanical characterization provided evidence concerning the effects of recycling and collagen peptide incorporation on the material’s tensile and compressive behavior. The recycling does not significantly compromise the PCL matrix, preserving its tensile and compressive properties to a large extent. At the same time, the collagen peptides enhance the tensile stiffness through molecular interactions while slightly reducing compressive strength. These findings suggest that the *PCL* + *Collagen peptides* filament can be a promising bioactive material in applications requiring tensile properties without significantly sacrificing mechanical integrity.

### Printability of *PCL* + *Collagen peptides* triply periodic minimal surface (TPMS)-based structures

**Fig. 8:**
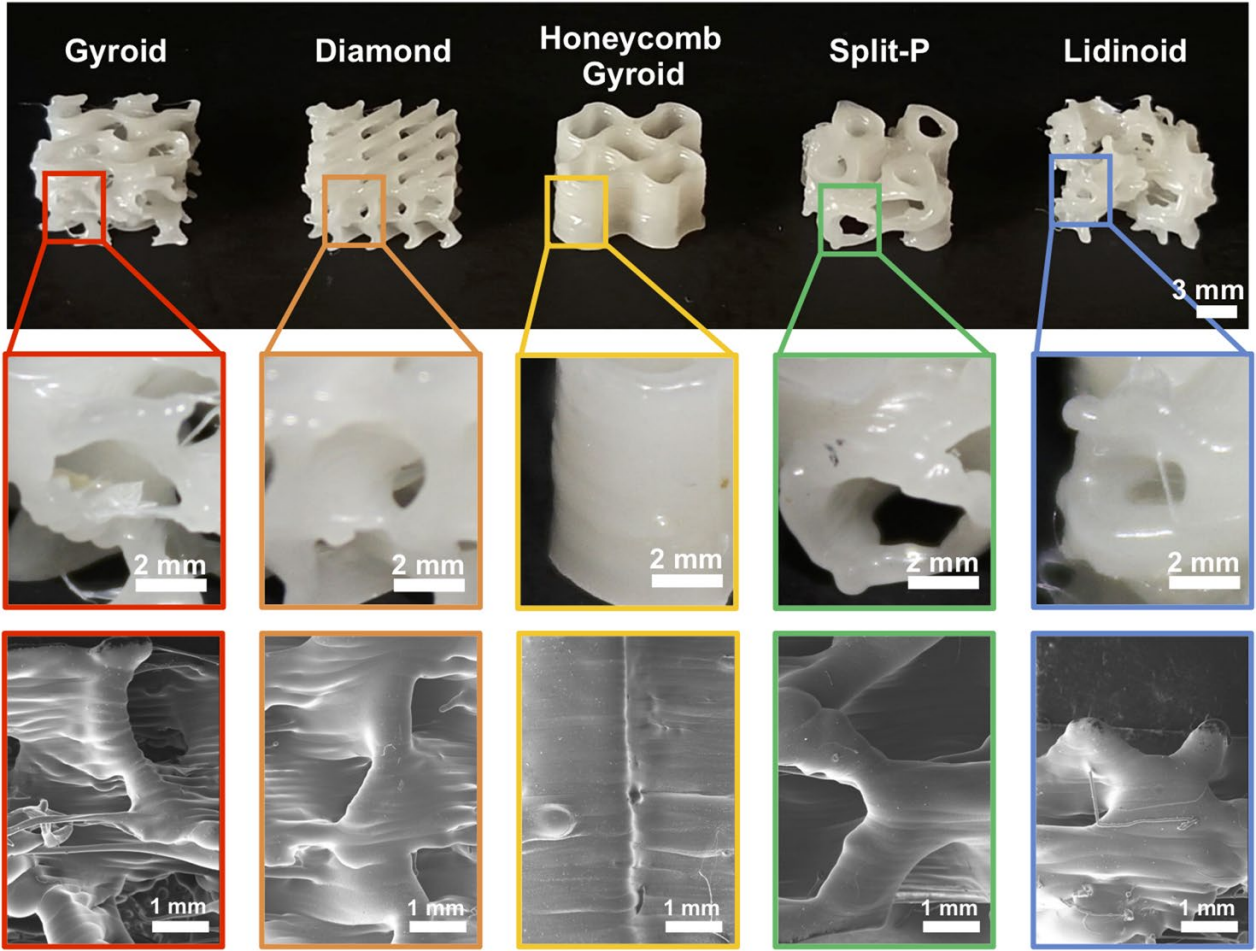
3D-printed TPMS structures using the *PCL* + *collagen peptides* filament. Five types of unit cells are shown (from left to right): Gyroid, Diamond, Honeycomb Gyroid, Split-P, and Lidinoid. The macroscopic images (top row) display the overall geometry, while the optical close-ups (middle row) highlight the internal surface details. SEM micrographs (bottom row) confirm the high printing quality.

Figure 8 shows the Gyroid, Diamond, Honeycomb Gyroid, Split-P, and Lidinoid, TPMS-based structures printed using the *PCL* + *Collagen peptides* filament. An initial visual inspection revealed that the printing parameters used produced satisfactory results (Fig. 8). This outcome was noteworthy, given the experimental nature of the *PCL* + *collagen peptides* filament and the complexity and small size of the printed structures. Upon closer examination of the lattice surfaces, the infill appeared sufficiently uniform, with only minimal debris or stringing inside some of the structures. Moreover, SEM analyses corroborated these findings, revealing no visible voids in the sidewalls or other layer irregularities, as well as no defects such as sagging or poor layer adhesion (Fig. 8 and Supplementary Fig. S7).

Overall, these results demonstrate the feasibility of fabricating geometrically complex TPMS scaffolds using a collagen-enriched PCL filament. The successful printing of such architectures confirms that this material can ensure structural integrity and high printability, despite its lab-scale and experimental nature, and that this property could be exploited for the development of customizable scaffolds with tunable architectures for tissue engineering applications.

## Conclusions

This study successfully developed a novel 3D-printable filament for FFF 3D printing by integrating collagen peptides into a PCL matrix through a non-toxic, solvent-assisted blending process and an optimized filament extrusion process. Additionally, the influence of a potential recycling process on the production of the PCL matrix has been investigated, and dedicated filaments have been fabricated. The study focused on developing new 3D printable filaments, rather than pellets, because we aimed to demonstrate the possibility of creating raw materials suitable for an additive manufacturing technology (the FFF) that is not only affordable but also widely accessible. The fact that 3D-printable filament-based materials can be obtained, thanks to the developed protocol, further strengthens the study’s potential impact.

Structural, morphological, and mechanical characterizations confirmed that incorporating collagen peptides preserves the PCL matrix and provides bioactive functionalities. Through X-ray diffraction, intrinsic fluorescence, DSC, and TGA, we demonstrated that the collagen peptides influence the crystallinity and thermal behavior of PCL. The mechanical testing on 3D-printed samples revealed that recycling does not significantly compromise the tensile and compressive properties of PCL; moreover, the addition of collagen enhances tensile stiffness due to hydrogen bonding between the collagen’s functional groups and the PCL matrix. Degradability studies have also revealed the possibility of tuning this behavior by working at the matrix level and through the presence of collagen peptides. Biocompatibility studies have demonstrated that this characteristic of the matrix is preserved even in the presence of the collagen peptides. Additionally, the possibility of using the novel bioactive composite filament to 3D print complex shapes, such as TPMS-based scaffolds, has also been demonstrated.

Therefore, all these results confirm that this new type of functionalized composite material could represent a sustainable and cost-effective alternative for various biomedical applications, from patient-specific implants to tissue scaffolds and wound healing devices. Indeed, it is not by chance that affordable technologies have been utilized to develop it, both for creating the raw material (i.e., the filament) and for utilizing it in the manufacturing of artifacts. Future research may refine the composite formulation to enhance bioactivity and in *vitro* performance, investigate in vivo functionalities, and incorporate additional bioactive molecules. Besides, to support future industrial translation, it will be essential to source collagen peptides from commercially available suppliers who can certify raw material origin and processing, and ensure a consistent peptide profile (< 3 kDa, which can exert biological activities); such products are already used in the food and nutraceutical industries, where strict quality control standards help guarantee batch-to-batch reproducibility.

## Supplementary Information

Below is the link to the electronic supplementary material.

Supplementary Material 1

## Data Availability

The datasets generated during and/or analysed during the current study are available from the corresponding authors upon reasonable request.
