# Supplementary material for: Development of a 3D-printable bioactive polycaprolactone–collagen peptides filament for biomedical applications
Source: Sci Rep. 2025 Dec 24;15:44513. doi: 10.1038/s41598-025-28030-5 (PMC12738878; doi:10.1038/s41598-025-28030-5)
Supplement: Supplementary file 1 — Supplementary Material 1 [file 41598_2025_28030_MOESM1_ESM.pdf]

Supplementary information for

Development of a 3D-printable bioactive polycaprolactone–collagen peptides filament for biomedical applications

Stefano Cantella[1], Silvia Badini[2], Carlotta Bollati[3], Mushtaq Alam Madar Saheb[4], Roberto Viganò[4], Carmen Lammi[3], Raffaele Pugliese[2,*] & Serena Graziosi[4,*]

[1]School of Industrial and Information Engineering, Politecnico di Milano, 20133, Milan, Italy.
[2]NeMO Lab Research Center, ASST GOM Niguarda Cà Granda Hospital, 20152, Milan, Italy.
[3]Department of Pharmaceutical Sciences, University of Milan, 20133, Milan, Italy
[4]Department of Mechanical Engineering, Politecnico di Milano, 20156, Milan, Italy

[*] Corresponding author at: serena.graziosi@polimi.it (SG), raffaele.pugliese@nemolab.it (RP)

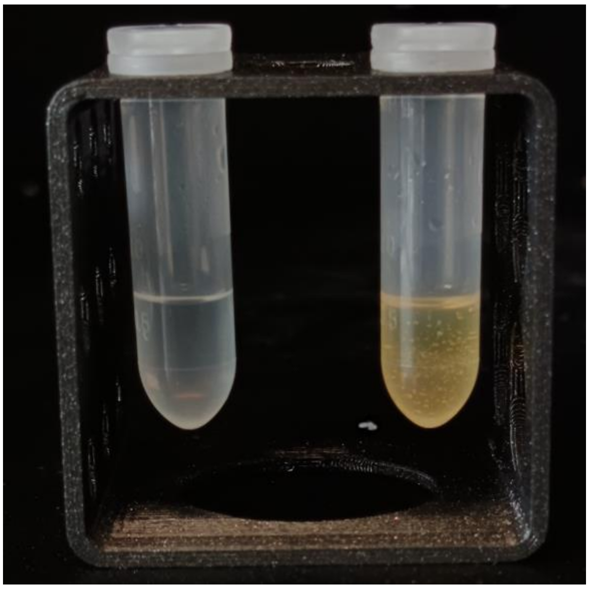

Supplementary Figure S1. Dissolution behavior of collagen peptides in a 95:5 (v/v) mixture of isopropyl alcohol (IPA) and double-distilled water (ddH_2O). The 1.5% (w/v) collagen peptide solution exhibits complete dissolution, leading to a clear, homogeneous mixture (on the left). A 3% (w/v) collagen peptide concentration leads to hydrogel formation, making it unsuitable for incorporation into PCL bits (on the right).

Supplementary Table S1. Felfil extrusion parameters provided by the manufacturer[1].

Material	Extruder Nozzle Diameter [mm]	Extruder Mode	Extrusion Temperature [°C]	Screw Speed [rpm]
PCL	1.75	Soft	95 °C	8 rpm

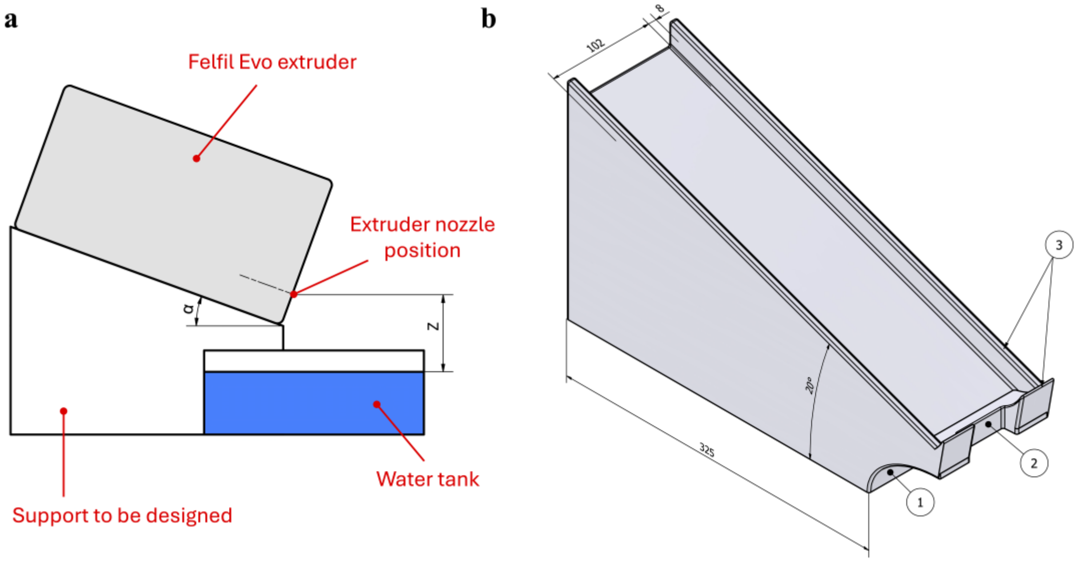

Supplementary Figure S2. Custom-designed support for the extruder. **(a)** Schematic representation of the support, highlighting its 20° tilting angle (α), the relationship between the nozzle position, and its distance (z) from the water surface. **(b)** Rendering of the implemented support, where (1) is a bottom cavity beneath the support's end to accommodate the water tank, ensuring water contact well away from the tank edges. (2) is a centrally located opening providing an unobstructed path for the filament to fall directly into the water tank. (3) indicates the perimeter walls to hold the extruder in place during operation. To optimize printing times and material usage, the support has not been printed as fully solid, but as represented in **Fig. 1**.

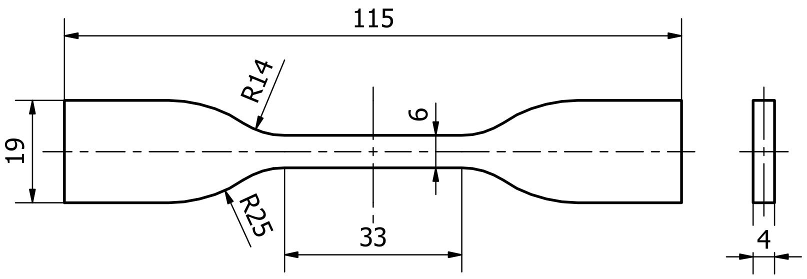

Supplementary Figure S3. The dimensions of the tensile sample according to the ASTM D638-14 standard.

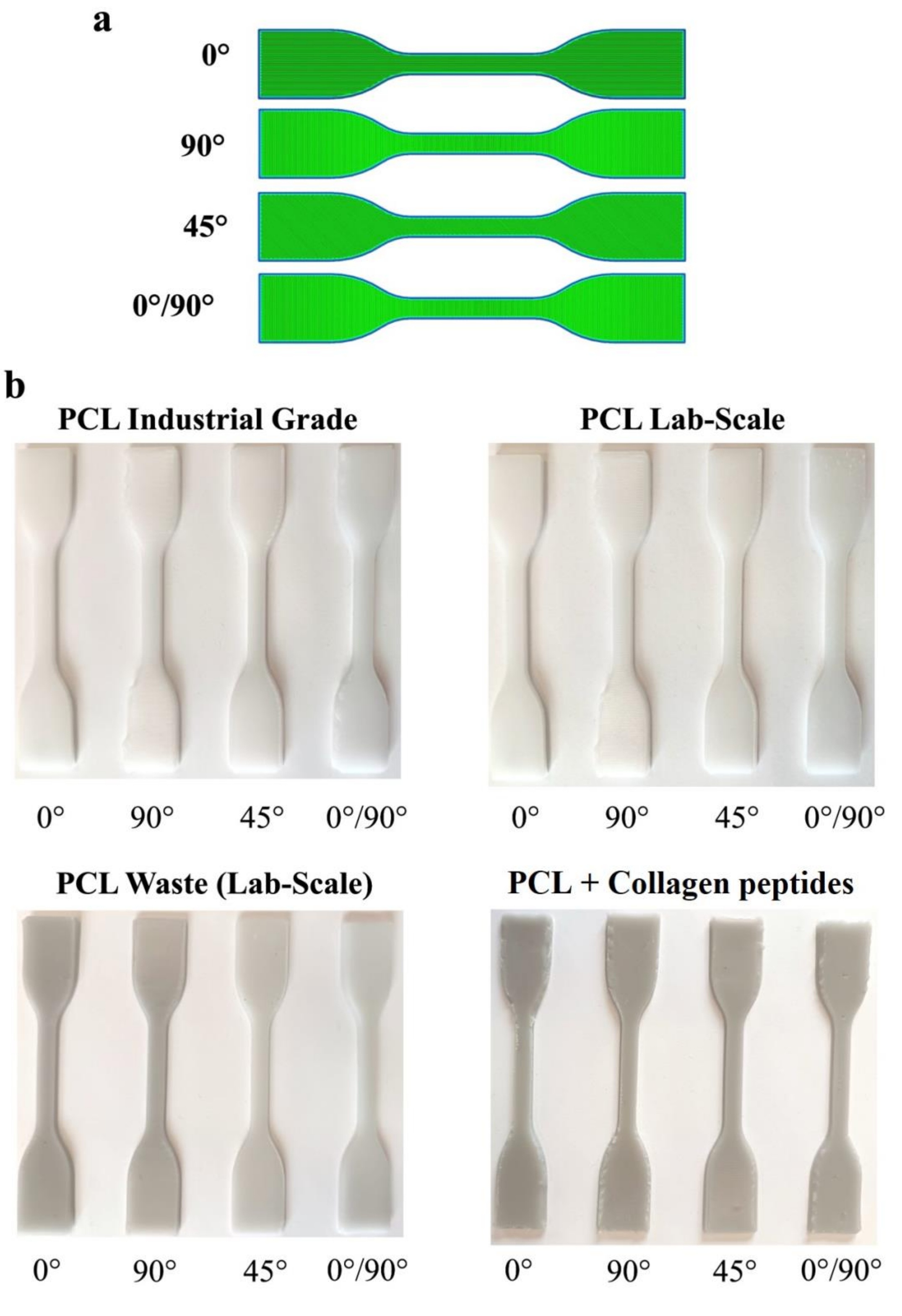

Supplementary Figure S4. The 3D-printed tensile samples. **(a)** Overview of the selected infill orientations. **(b)** Overview of the various 3D-printed samples for each type of material analyzed in the study (i.e., *PCL Industrial Grade, PCL Lab-Scale, PCL Waste (Lab-Scale), PCL + Collagen peptides*).

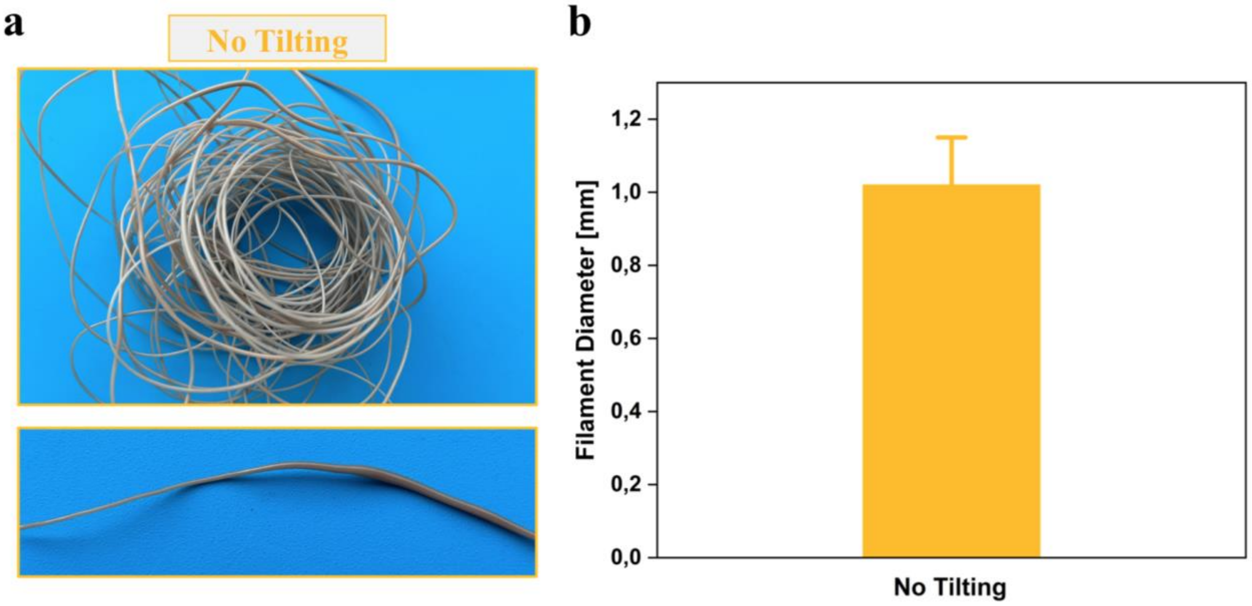

Supplementary Figure S5. Examples of issues observed during the filament manufacturing when no optimization is applied. **(a)** Extruded defective filament without the time-of-flight (ToF) and the 20° tilting angle (α) optimization. **(b)** The diameter obtained for this filament is 1.02 ± 0.13 mm, which is significantly lower than that of commercial filaments (i.e., 1.75 mm).

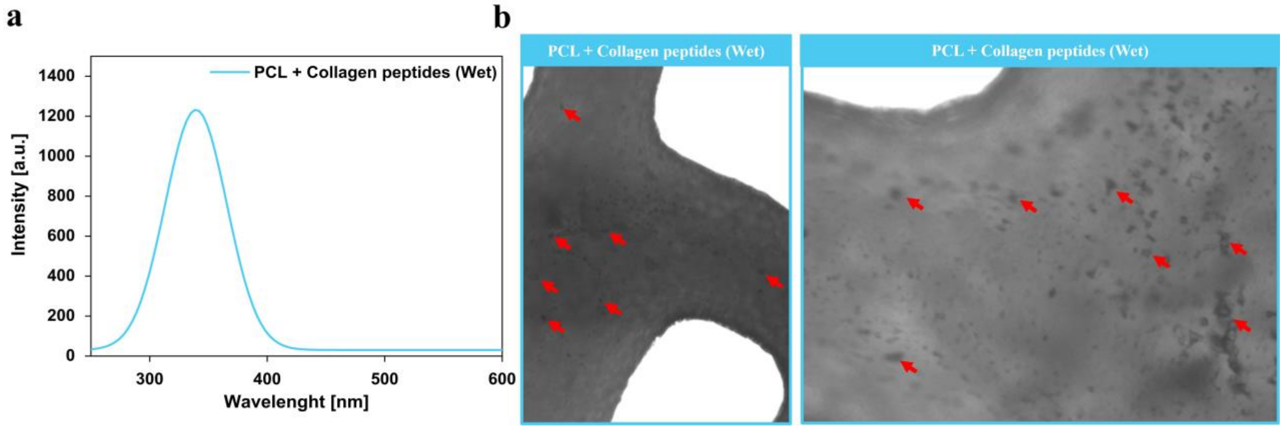

Supplementary Figure S6. A confirmation of the presence of collagen peptides within the matrix. **(a)** Fluorescence behavior of *PCL + Collagen peptides* composite after 96-hour immersion in an aqueous environment. The composite maintained fluorescence intensity, indicating the sustained presence of collagen peptides. **(b)** Optical microscopy images further confirmed the retention of collagen peptides within the PCL matrix post-immersion.

Supplementary Table S2. Average values of the tensile modulus and peak stress along all raster angles of the PCL-based materials.

Material	Raster Angle [°]	Average Tensile Modulus [MPa]	Average Peak Stress [MPa]
PCL Industrial Grade	0°	570.60±54.59	21.15±0.11
	90°	576.94±26.65	21.60±0.22
	45°	544.00±35.32	21.34±0.22
	0°/90°	564.00±36.88	20.83±0.24
PCL Lab-Scale	0°	475.87±37.05	18.25±0.60
	90°	502.83±30.45	17.98±1.02
	45°	441.63±44.56	18.39±0.30
	0°/90°	494.23±25.92	18.09±0.41
PCL Waste (Lab-Scale)	0°	514.30±23.08	20.14±1.39
	90°	498.99±21.68	20.19±0.24
	45°	474.46±16.50	18.31±0.17
	0°/90°	465.40±45.42	18.86±0.82
PCL + Collagen peptides	0°	528.53±27.15	18.73±0.36
	90°	553.25±40.17	18.30±1.8
	45°	526.11±31.97	19.12±0.40
	0°/90°	534.36±19.42	17.97±0.56

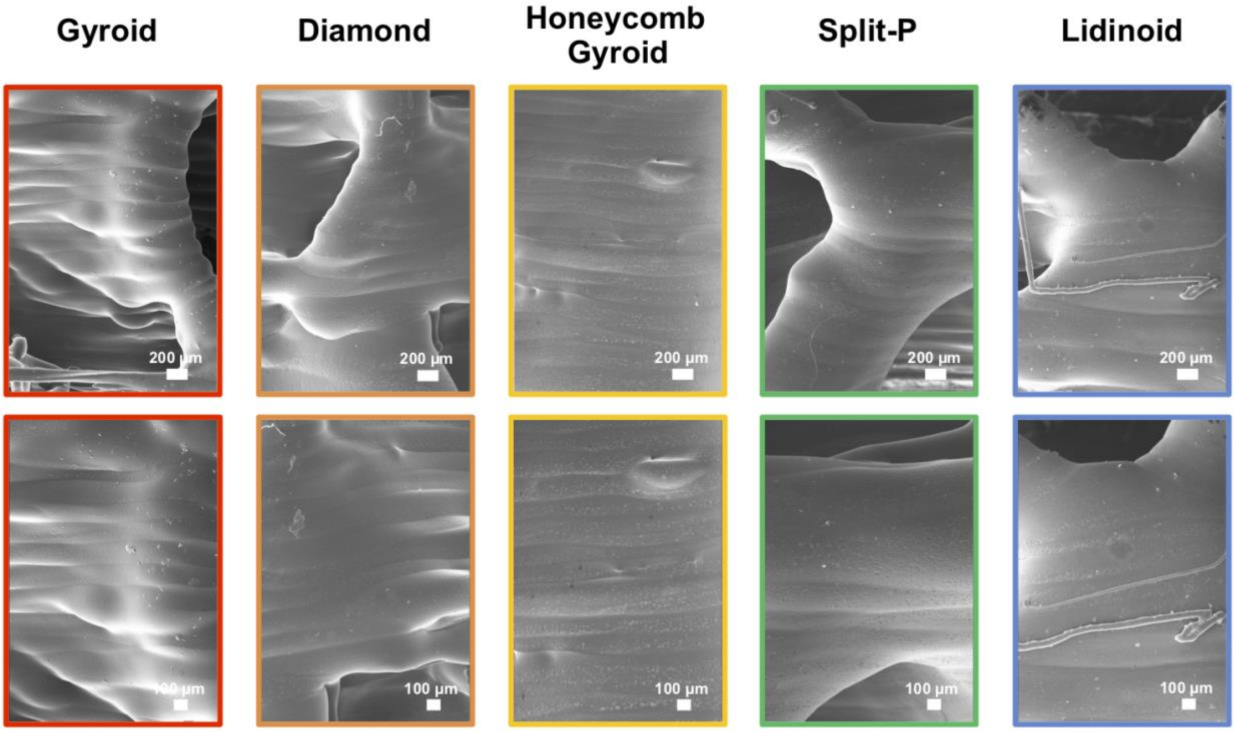

Supplementary Figure S7. SEM micrographs of 3D-printed TPMS-based structures using the *PCL + collagen peptides* filament. Representative views of Gyroid, Diamond, Honeycomb Gyroid, Split-P, and Lidinoid unit cells are shown at two magnifications. The images reveal smooth sidewalls with no visible voids, cracks, or delamination, further confirming the good interlayer adhesion and the high printing quality achieved with the optimized parameters.

Supplementary References

1. Felfil, Guide: extruding the most common polymers with Felfil System, available at https://felfil.com/guide-extruding-the-most-common-polymers-with-felfil-system/?v=058f38ac933, last accessed April 7, 2025.
